# Association between neonatal birthweight and maternal vaginal wall prolapse: a retrospective analysis of postpartum women

**DOI:** 10.3389/fmed.2026.1792690

**Published:** 2026-03-12

**Authors:** Zhuwei Gao, Yuan Wang, Lichi Zhang, Hongxian Li, Aiju Chen, Yue Yuan, Xinghui Chen

**Affiliations:** 1 Graduate School, Heilongjiang University of Chinese Medicine, Harbin, Heilongjiang, China; 2 Pelvic Floor Rehabilitation Medical Center, Kunming Maternal and Child Health Hospital, Kunming, Yunnan, China

**Keywords:** neonatal birthweight, pelvic organ prolapse, posterior vaginal wall prolapse, risk factors, postpartum assessment

## Abstract

**Objective:**

To investigate the association between neonatal birthweight (BW) and the maternal risk of anterior and posterior vaginal wall prolapse in the postpartum period.

**Methods:**

This retrospective cross-sectional study included 864 postpartum women undergoing routine follow-ups after delivery. Participants were categorized into quartiles according to BW. Multivariate logistic regression models were applied to evaluate the association between BW and moderate-to-severe vaginal wall prolapse [Pelvic Organ Prolapse Quantification (POP-Q), POP-Q stage ≥ II], sequentially adjusting for maternal age, parity, mode of delivery, and plurality. Restricted cubic spline (RCS) analyses were used to examine potential non-linear relationships, accompanied by subgroup and sensitivity analyses.

**Results:**

In the fully adjusted model, each 1 kg increase in BW was associated with a 71% higher risk of posterior vaginal wall prolapse (OR = 1.71, 95% CI: 1.18–2.53, *P* = 0.006), whereas the overall association with the anterior wall was not significant (OR = 1.35, 95% CI: 0.93–1.98, *P* = 0.125). In the quartile analysis, women in the highest BW group (≥3.415 kg) had a 2.26-fold higher risk of posterior wall prolapse (*P* for trend < 0.001). In comparison, a significant increase in risk was also observed for the anterior wall in the moderate BW group (3.15–3.415 kg) (OR = 1.62, 95% CI: 1.02–2.61). RCS analysis revealed non-linear dose–response relationships between BW and anterior and posterior wall prolapse, with inflection points around 3.2 kg and 3.7 kg, respectively. The risk increase was more pronounced in the lower BW range (< inflection point), and delivery mode acted as a significant effect modifier.

**Conclusion:**

Higher BW is an independent risk factor for maternal posterior vaginal wall prolapse, showing both dose-dependent and threshold effects. The results suggest that women who deliver larger babies may require enhanced postpartum pelvic floor evaluation and follow-up management, and further prospective studies are needed to explore the long-term implications of these findings on pelvic floor health.

## 1 Introduction

Pelvic organ prolapse (POP) refers to the descent of one or more pelvic organs (such as the uterus, bladder, or rectum) from their normal anatomical positions, sometimes even protruding beyond the vaginal introitus. It is classified as a pelvic floor dysfunction (PFDs) (1). Anterior vaginal wall prolapse, posterior vaginal wall prolapse, and apical prolapse are the core subtypes of POP. Among them, anterior vaginal wall prolapse occurs approximately twice as often as posterior vaginal wall prolapse and three times as often as apical prolapse, making it the most common clinical form of POP (2). POP not only impairs women's physical health but also significantly affects their quality of life, sexual function, and psychological wellbeing. With the global trend of population aging and rising awareness of life quality, POP has emerged as a growing public health concern (3).

The etiology of POP is multifactorial, involving complex interactions between genetic predisposition and environmental influences (4). Established risk factors include advancing age, multiparity, obesity or overweight, chronically elevated intra-abdominal pressure, history of pelvic surgery, and hereditary susceptibility (5). Among the numerous obstetric variables, BW represents a significant mechanical load on the maternal pelvic floor during delivery. Theoretically, a higher BW leads to greater stretching and prolonged strain on pelvic floor muscles—particularly the levator ani—and connective tissues during vaginal delivery, thereby increasing the likelihood of structural injury (6). Such injuries may manifest as levator ani avulsion, excessive muscle fiber stretching, nerve damage, and fascial tearing (7). The cumulative effect of these structural damages may weaken pelvic support function, predisposing women to the development of POP.

However, the evidence linking higher BW to POP and urinary incontinence (UI) remains inconsistent (8, 9). Moreover, few studies have specifically examined the association between BW and compartment-specific prolapse (such as anterior or posterior vaginal wall descent) in Chinese women. Most existing studies have treated POP as a single composite outcome or have failed to adequately adjust for key confounders such as parity and delivery mode. In addition, pelvic floor tissues in the early postpartum period may still exhibit a certain degree of physiological laxity and reversible anatomical changes; therefore, defining clinically relevant prolapse and assessing its risk require a clear and standardized outcome definition.

Therefore, this study aimed to explore the independent associations between BW and the risks of moderate-to-severe anterior and posterior vaginal wall prolapse in Chinese postpartum women using a rigorous multivariate statistical framework. The findings are expected to provide evidence for identifying high-risk populations and developing targeted preventive strategies in clinical practice.

## 2 Methods

### 2.1 Study population

This study was a retrospective cross-sectional study. The research data were obtained from the electronic medical record system of the Pelvic Floor Rehabilitation Center of Kunming Maternal and Child Health Hospital, where postpartum pelvic floor assessment at 42 days is routinely included as part of standard postnatal follow-up. The system records standardized examination data of women who undergo postpartum assessments or seek pelvic floor-related care. 879 women were initially enrolled; after excluding 15 cases with missing BW data, 864 participants were included in the final analysis. Participants were categorized into quartiles based on BW.

### 2.2 Inclusion and exclusion criteria

Inclusion criteria: (1) Chinese postpartum women aged ≥20 years; (2) gestational age ≥ 37 weeks with live birth; (3) underwent pelvic floor examination and completed standardized POP-Q assessment during postpartum follow-up; (4) complete medical records and follow-up data, including delivery mode and BW.

Exclusion criteria: (1) preexisting POP or other severe PFDs confirmed by gynecologic examination before pregnancy; (2) systemic diseases affecting connective tissue or muscle function; (3) history of pelvic surgery or severe pelvic trauma; (4) underwent non-gynecologic surgery during the study period that might affect pelvic floor structure.

### 2.3 Data collection and preprocessing

All data were retrieved from existing electronic medical records. Information collected included baseline characteristics (age, parity, gestational weeks, and pregnancy complications), delivery-related variables (delivery mode, perineal condition, and BW), and standardized POP-Q assessment records completed at the 42-day postpartum visit. For non-perinatal data, POP-Q standardized assessment records measured during the clinical visit were used. Before conducting statistical analysis, we pre-processed the data. Missing data were handled using listwise deletion. Missing data were handled by listwise deletion, as the missing rate of all variables was extremely low (0.1%~2.1%) and verified to be completely random (MCAR). This approach was selected for its simplicity and ability to avoid biases such as variance underestimation or overfitting associated with imputation methods, ensuring data authenticity and result reliability.

### 2.4 Main variables

#### 2.4.1 Outcome variables

POP was objectively evaluated using the POP-Q system (10). All examinations were performed by a single pelvic floor specialist with extensive clinical experience who had received standardized training and were conducted in strict accordance with the standardized examination protocol recommended by the International Continence Society (ICS) (11). Examinations were performed in the lithotomy position with the bladder moderately distended, following the ICS-recommended measurement sequence and procedures. All POP-Q landmarks were measured using a marked ruler to the nearest 0.5 cm during maximal Valsalva effort to minimize measurement variability and ensure procedural consistency. Reference points on the anterior vaginal wall, cervix, and posterior vaginal wall (Aa, Ba, C, Ap, Bp) were used to measure the extent of prolapse, with the hymen set as the zero reference point for vertical distance recording. The POP-Q stages were defined as follows: stage 0, no prolapse; stage I, the lowest point > 1 cm above the hymen; stage II, the lowest point ≤ 1 cm above or below the hymen; stage III, prolapse extending ≥ 1 cm beyond the hymen; and stage IV, complete eversion.

To ensure adequate sample size and statistical stability, prolapse of stage II or higher (POP-Q ≥ II) was defined as a positive outcome, including “anterior vaginal wall prolapse ≥ II” and “posterior vaginal wall prolapse ≥ II”. The anterior wall mainly represents cystocele, and the posterior wall corresponds to rectocele. POP-Q stage ≥ II was selected as the definition of clinically relevant prolapse in the present study. Previous studies have shown that when vaginal wall descent approaches the hymenal plane, patient-reported awareness of vaginal bulge or protrusion increases substantially, and descent to 0.5 cm distal to the hymenal remnant identifies bulge symptoms with a sensitivity of up to 97% (12). In contrast, when the leading edge of prolapse remains at or above the hymenal plane, most women are typically asymptomatic, suggesting that milder anatomical descent may reflect physiological variation rather than clinically meaningful prolapse (13). In addition, imaging studies have demonstrated that prolapse located within 1 cm of the hymenal plane is more likely to be accompanied by levator ani muscle injury, indicating that this anatomical level more closely corresponds to substantive impairment of pelvic floor support structures (2). Taken together, the use of POP-Q stage ≥ II allows for a more appropriate distinction between clinically relevant prolapse and early, low-burden anatomical changes (Supplementary Figure S1, Supplementary Tables S1, S2). In addition, using POP-Q stage ≥ II at 42 days postpartum allows for a more sensitive identification of early pelvic support changes during the recovery period. However, it may also capture some transient physiological laxity rather than established long-term prolapse.

#### 2.4.2 Main exposure variable

The primary exposure variable was BW. To ensure measurement consistency, the recorded BW was used directly for singleton pregnancies; for multiple pregnancies, the highest BW among the neonates was selected; and for women with multiple deliveries, the BW of the most recent delivery was used as the exposure value. This definition reflects the maternal pelvic floor's most recent mechanical load and recovery status, minimizing recall bias or heterogeneity related to earlier deliveries.

#### 2.4.3 Covariates

**Figure 1:**
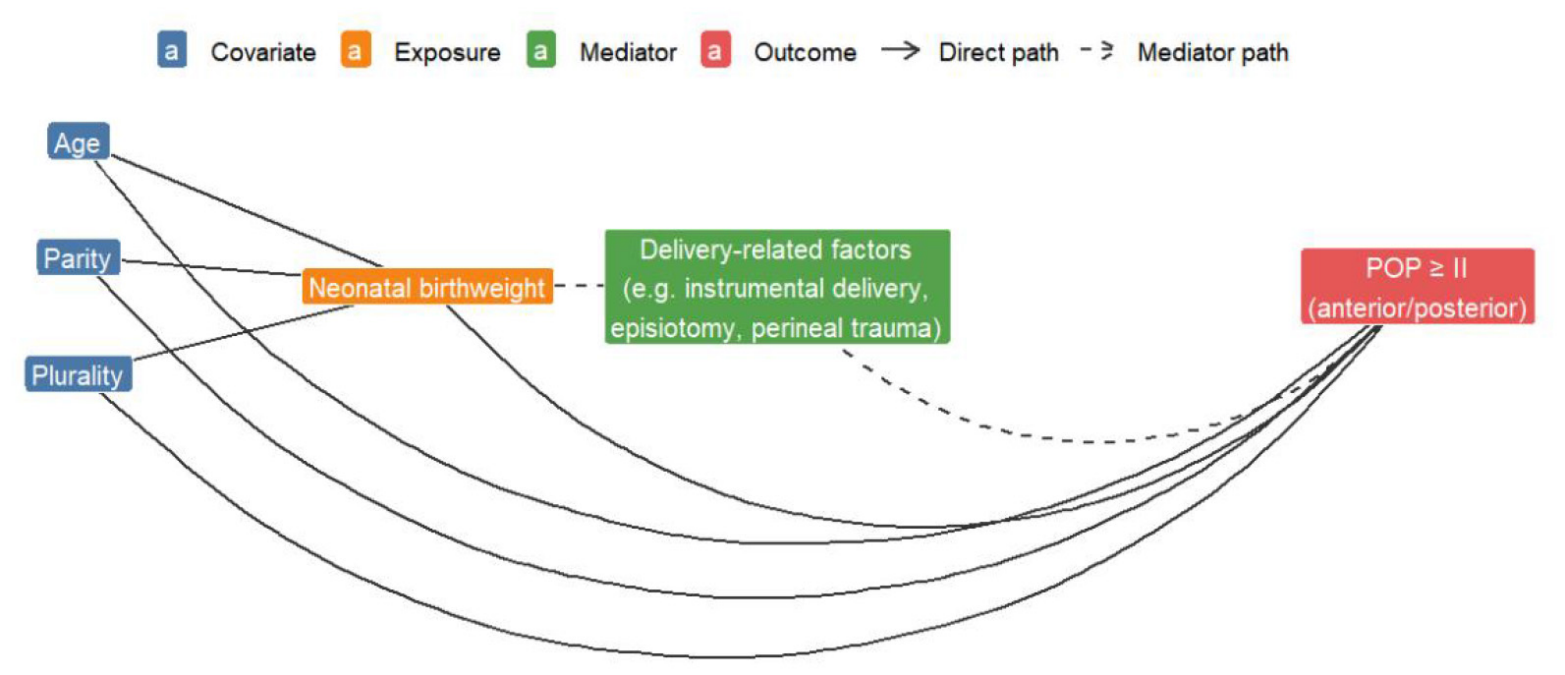
Causal DAG used for covariate selection.

Based on prior research and clinical evidence, potential confounders were systematically identified and controlled using a Directed Acyclic Graph (DAG) (Figure 1). Covariate selection included variables associated with POP outcomes and potentially causally related to the main exposure (BW), forming the minimal sufficient adjustment set. All covariates were derived from standardized questionnaires and medical records, defined as age (years): age at the time of assessment, included as a continuous variable and used for subgroup stratification. Parity: the number of deliveries, self-reported and verified against obstetric records, and treated as a count variable. Delivery mode: categorized as vaginal delivery or cesarean section. Plurality: categorized as singleton or twin/multiple pregnancy. To ensure the scientific rigor and reproducibility of covariate selection, we further summarized the evidence-based rationale for each potential confounder in relation to the risk of anterior and posterior vaginal wall prolapse based on findings from previous systematic reviews and observational studies (Supplementary Table S3).

### 2.5 Statistical analysis

All statistical analyses were conducted using R version 4.3.2 (R Foundation for Statistical Computing, Vienna, Austria) and the RStudio platform. Continuous variables were tested for normality using the Shapiro–Wilk test. Variables with a normal distribution were expressed as mean ± standard deviation and compared between groups using the independent-samples *t*-test (with Welch's correction applied when variances were unequal). Non-normally distributed variables were expressed as median (interquartile range) and compared using the Mann–Whitney U test. Categorical variables were presented as frequency (%), and differences between groups were assessed using the chi-square test or Fisher's exact test as appropriate.

Subsequently, binary logistic regression models were employed to evaluate the association between BW and the risk of moderate-to-severe anterior or posterior vaginal wall prolapse (POP-Q ≥ II), with results presented as odds ratios (ORs) and 95% confidence intervals (CIs). Three models were constructed according to the degree of covariate adjustment: the unadjusted model (including only the primary exposure variable), the partially adjusted model (further adjusting for age and parity), and the fully adjusted model (additionally adjusting for delivery mode and plurality). To explore the non-linear relationship between BW and anterior/posterior vaginal wall prolapse, this study constructed a RCS regression model based on the covariate set from the fully adjusted model. The specific parameter settings were as follows: nodes were placed at the 5th, 35th, 65th, and 95th percentiles of singleton birth weight distribution, balancing model flexibility and statistical efficiency; the reference point was set at the median of birth weight, and the odds ratio (OR) and 95% confidence interval (CI) were calculated for the adjusted estimates. Non-linearity was tested using the likelihood ratio test (LRT) by comparing the fit of the RCS model with that of a linear regression model, further validating the statistical significance of the non-linear relationship. Additionally, we used a segmented regression model to verify potential threshold effects. Based on the RCS model fitting results, the optimal breakpoint was estimated using the maximum likelihood method; the data were then split at this breakpoint, and linear regression models were fit for each of the two segments, calculating the respective regression coefficients (slopes). Finally, the LRT was applied to compare the fit of the segmented regression model with the simple linear regression model, to verify the presence of the threshold effect.

Subgroup analyses were performed by age, delivery mode, plurality, and parity, and forest plots were generated to display interaction effects (*P* for interaction). To test the robustness of the results, several sensitivity analyses were conducted, including redefining the outcome threshold as POP-Q ≥ I and repeating analyses in specific subgroups, such as women with singleton vaginal deliveries (Supplementary Tables S4, S5). All statistical tests were two-sided; a *P* value < 0.05 was considered statistically significant.

## 3 Results

### 3.1 Participant characteristics

A total of 879 participants were included in this study, of whom 15 had missing data on neonatal BW. Among the 864 participants with complete BW data, participants were grouped into four quartiles (Q1–Q4) based on neonatal BW for descriptive analyses, with sample sizes of 228, 210, 211, and 215 in each quartile, respectively. The median age was comparable among the four groups (*P* = 0.995), and parity did not differ significantly (*P* = 0.175). Singleton pregnancies predominated across all groups, with no significant difference in plurality (*P* = 0.097).

Regarding delivery mode, the overall vaginal delivery rate was 82.98% (702/846). The rates across Q1–Q4 were 77.83%, 85.37%, 85.17%, and 83.89%, respectively, showing no significant difference (*P* = 0.121). The rate of instrumental delivery was 2.7% overall, with no significant difference across the four groups (*P* = 0.508).

During pregnancy, the overall prevalence of UI was 24.0%, with rates of 28.9%, 24.3%, 27.0%, and 10.2% across the four groups, showing no significant difference (*P* = 1.00). The overall prevalence of pelvic or lumbosacral pain was 12.5%, with group-specific rates of 13.2%, 14.8%, 11.8%, and 10.2%, without a significant difference (*P* = 1.00).

During delivery, the incidence of episiotomy was 28.7%, with rates of 21.9%, 31.0%, 32.2%, and 30.2% across Q1-Q4, with no significant difference among groups (*P* = 0.067). The incidence of perineal laceration was 45.6%, with rates of 39.9%, 45.2%, 49.8%, and 47.9%, also showing no significant difference (*P* = 0.171) (Table 1).

**Table 1:** Baseline and perinatal characteristics across BW quartiles.

**Variable (unit, summary type)**	**Total**	**Q1 (Low)**	**Q2**	**Q3**	**Q4 (High)**	***P***
No. of participants (*n*)	864	228	210	211	215	
Age (years), median (IQR)	29.00 (26.00,31.00)	29.00 (26.00,31.00)	29.00 (26.00,31.00)	29.00 (26.00,32.00)	29.00 (27.00,31.00)	0.995
Parity, median (IQR)	1.00 (1.00,2.00)	1.00 (1.00,2.00)	1.00 (1.00,2.00)	1.00 (1.00,2.00)	1.00 (1.00,2.00)	0.175
**Plurality**, ***n*** **(%)**						0.097
Singleton	861 (99.3%)	205 (98.1%)	213 (99.5%)	221 (100%)	213 (99.5%)	
Twin	6 (0.7%)	4 (1.9%)	1 (0.5%)	0 (0%)	1 (0.5%)	
**Delivery mode**						0.121
Vaginal spontaneous delivery	702 (82.98%)	172 (77.83%)	175 (85.37%)	178 (85.17%)	177 (83.89%)	
Cesarean section	144 (17.02%)	49 (22.17%)	30 (14.63%)	31 (14.83%)	34 (16.11%)	
Instrumental delivery, *n* (%)	23 (2.7%)	3 (1.3%)	7 (3.3%)	7 (3.3%)	6 (2.8%)	0.508
**Conditions during pregnancy**						
Urinary incontinence (yes), *n* (%)	207 (24.0%)	66 (28.9%)	51 (24.3%)	57 (27.0%)	33 (10.2%)	1.00
Pelvic/lumbosacral pain (yes), *n* (%)	108 (12.5%)	30 (13.2%)	31 (14.8%)	25 (11.8%)	22 (10.2%)	1.00
**Intrapartum conditions**						
Episiotomy, *n* (%)	248 (28.7%)	50 (21.9%)	65 (31.0%)	68 (32.2%)	65 (30.2%)	0.067
Perineal laceration, *n* (%)	394 (45.6%)	91 (39.9%)	95 (45.2%)	105 (49.8%)	103 (47.9%)	0.171

All percentages are calculated based on the valid sample size. A small number of missing values exist for some variables (e.g., 18 cases with missing delivery mode, 11 cases with missing parity) (Supplementary Table S6).

### 3.2 Association between BW and the risk of anterior and posterior vaginal wall prolapse

When BW was analyzed as a continuous variable, the risk of posterior vaginal wall prolapse increased significantly with each 1 kg rise in BW: unadjusted model OR = 1.76 (95% CI: 1.27–2.51, *P* = 0.001); partially adjusted model OR = 1.73 (1.23–2.50, *P* = 0.002); and fully adjusted model OR = 1.71 (1.18–2.53, *P* = 0.006). For anterior vaginal wall prolapse, a positive association was also observed in the unadjusted model (OR = 1.51, 1.08–2.14, *P* = 0.019), but the association weakened after adjustment for age and parity (OR = 1.42, 1.00–2.06, *P* = 0.058) and became non-significant in the fully adjusted model (OR = 1.35, 0.93–1.98, *P* = 0.125).

When BW was analyzed as a categorical variable based on quartiles, the risk of posterior vaginal wall prolapse increased progressively with higher quartile groups: using Q1 (< 2.9 kg) as the reference, the fully adjusted model showed that women in Q4 (≥3.415 kg) had an OR of 2.26 (95% CI: 1.39–3.73), with a significant trend across all three models (*P* for trend < 0.001). The association with anterior vaginal wall prolapse exhibited a distinct non-linear pattern rather than a uniform weak trend: in the fully adjusted model, the moderate BW group (Q3, 3.15–3.415 kg) showed a significantly elevated risk (OR = 1.62, 95% CI: 1.02–2.61), while the highest BW group (Q4, ≥3.415 kg) was only marginally significant (OR = 1.53, 95% CI: 0.95–2.46), and the trend test approached significance (*P* for trend = 0.063). This suggests that the risk of anterior vaginal wall prolapse does not increase linearly across the entire BW spectrum but shows a specific elevation in the moderate BW range (Table 2). Sensitivity analyses using POP-Q stage ≥ I as the outcome showed similar directional patterns; however, the near-universal prevalence of POP-Q ≥ I limited risk discrimination (Supplementary Table S4).

**Table 2:** Association between BW and moderate-to-severe anterior/posterior vaginal wall prolapse across quartiles.

**Outcome**	**Model**	**BW (kg)**		**BW quartiles OR (95% CI)**				
		**OR (95% CI)**	***P***	**Q1 (**<**2.9 kg)**	**Q2 (2.9–3.15 kg)**	**Q3 (3.15–3.415 kg)**	**Q4 (**≥**3.415 kg)**	***P*** **for trend**
Anterior	POP-Q ≥ II cases, n			151	158	167	166	
	Unadjusted	1.51 (1.08–2.14)	0.019^*^	Reference	1.61 (1.04–2.51)	1.85 (1.19–2.89)	1.59 (1.03–2.47)	0.026^*^
	Partially adjusted	1.42 (1.00–2.06)	0.058	Reference	1.58 (1.01–2.46)	1.79 (1.15–2.80)	1.60 (1.03–2.51)	0.028^*^
	Fully adjusted	1.35 (0.93–1.98)	0.125	Reference	1.43 (0.90–2.30)	1.62 (1.02–2.61)	1.53 (0.95–2.46)	0.063
Posterior	POP-Q ≥ II cases, n			39	50	58	71	
	Unadjusted	1.76 (1.27–2.51)	0.001^*^	Reference	1.34 (0.83–2.17)	1.78 (1.13–2.85)	2.20 (1.40–3.49)	< 0.001^*^
	Partially adjusted	1.73 (1.23–2.50)	0.002^*^	Reference	1.35 (0.83–2.21)	1.71 (1.07–2.74)	2.23 (1.41–3.57)	<0.001^*^
	Fully adjusted	1.71 (1.18–2.53)	0.006^*^	Reference	1.33 (0.80–2.24)	1.62 (0.99–2.68)	2.26 (1.39–3.73)	< 0.001^*^

The outcome was defined as POP-Q stage ≥ II for anterior or posterior vaginal POP. ORs and 95% CIs were calculated from binary logistic regression models (Firth correction used when separation was detected, if available). *P* for trend were estimated using the BW quartile as an ordinal variable. Partially adjusted includes age and parity. Fully adjusted additionally includes the delivery mode and plurality. ^*^P < 0.05.

### 3.3 Dose–response and non-linear association between BW and moderate-to-severe anterior/posterior vaginal wall prolapse

**Figure 2:**
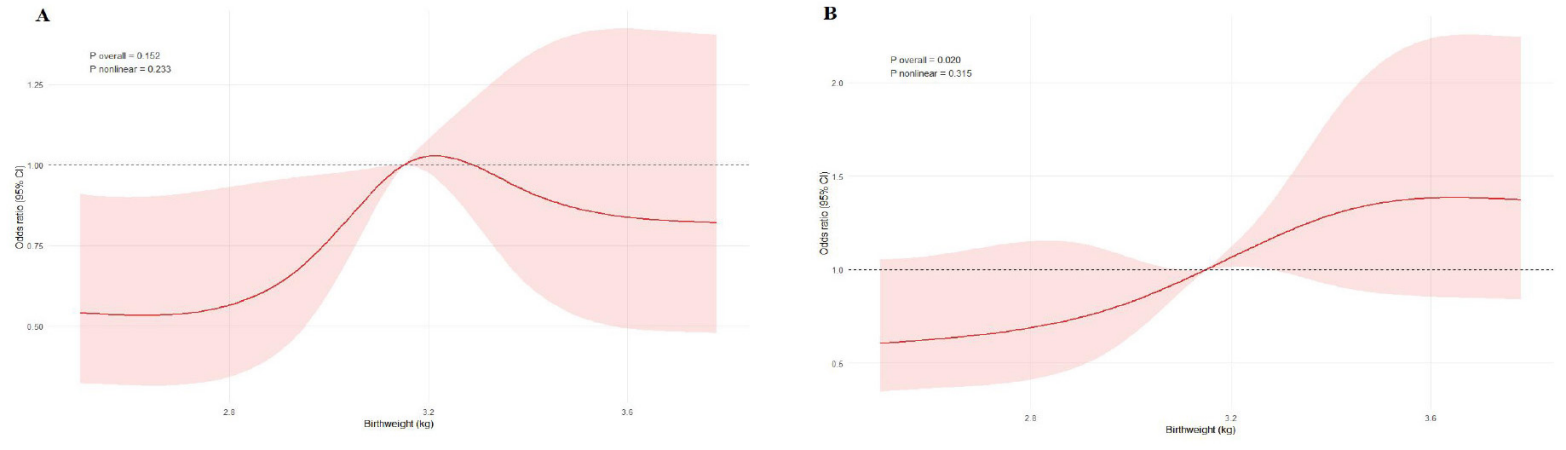
Dose–response relationship between BW and moderate-to-severe anterior and posterior vaginal wall prolapse. **(A)** RCS curve for moderate-to-severe anterior vaginal wall prolapse (fully adjusted). **(B)** RCS curve for moderate-to-severe posterior vaginal wall prolapse (fully adjusted).

RCS analysis further validated the results of the aforementioned quartile analysis, confirming a non-linear dose–response relationship between BW and POP quantified as POP-Q stage ≥ II. Notably, this non-linear association exhibited differences between the anterior and posterior vaginal walls (Figures 2A, B).

For the posterior vaginal wall (Figure 2B), the RCS curve showed an overall upward trend, with a steeper slope in the moderate-to-high BW range. Additionally, the CI remained above the reference line (odds ratio [OR] = 1) over a longer BW span, indicating more robust evidence of a non-linear association. Threshold effect analysis identified an inflection point at approximately 3.7 kg: below this threshold (< 3.7 kg), each 1-kg increase in BW was associated with a significant elevation in prolapse risk (OR = 2.20, 95% CI: 1.38–3.51); however, the association became statistically non-significant above this threshold (≥3.7 kg, OR = 0.80, 95% CI: 0.35–1.81; LRT for threshold, *P* = 0.060). This finding is consistent with the quartile analysis result that “the risk of posterior vaginal wall prolapse increased progressively with higher quartile groups, with a significant trend across all models (*P* for trend < 0.001)”, and explains the dose–response basis for the significantly elevated risk in the highest BW group (Q4, ≥3.415 kg).

For the anterior vaginal wall (Figure 2A), the RCS curve exhibited a gentler slope with an overall upward tendency; however, the confidence interval overlapped with the OR = 1 horizontal line in most BW ranges, suggesting a relatively weaker association. The inflection point for the anterior wall was approximately 3.2 kg, which closely aligns with the upper bound of the moderate BW quartile (Q3, 3.15–3.415 kg): below this threshold (< 3.2 kg), each 1-kg increase in BW was associated with a significant risk elevation (OR = 1.82, 95% CI: 1.02–3.22), corresponding to the specific risk increase observed in the Q3 group; above 3.2 kg, the association was non-significant (OR = 0.90, 95% CI: 0.48–1.70; LRT, *P* = 0.187). This explains why the highest BW group (Q4) showed only a marginally significant risk, with no further elevation.

The above results were consistent with the threshold analysis using a segmented linear model (Table 3), collectively confirming that increases in BW within the lower range were more strongly associated with elevated risks of moderate-to-severe maternal vaginal wall prolapse. In contrast, the association with anterior wall prolapse was comparatively weaker.

**Table 3:** Threshold effect analysis of BW on moderate-to-severe anterior and posterior vaginal wall prolapse.

**Outcome**	**BW (kg)**	**OR (95% CI)**	***P***	***P*-value for the LRT**
Anterior	<3.2	1.82 (1.02–3.22)	0.041^*^	0.187
	≥3.2	0.90 (0.48–1.70)	0.748	
Posterior	<3.7	2.20 (1.38–3.51)	< 0.001^*^	0.060
	≥3.7	0.80 (0.35–1.81)	0.586	

The threshold effect was assessed using a piecewise linear logistic model, with the LRT choosing a single change point. ^*^P < 0.05.

### 3.4 Subgroup analysis of BW and moderate-to-severe anterior/posterior vaginal wall prolapse

**Figure 3:**
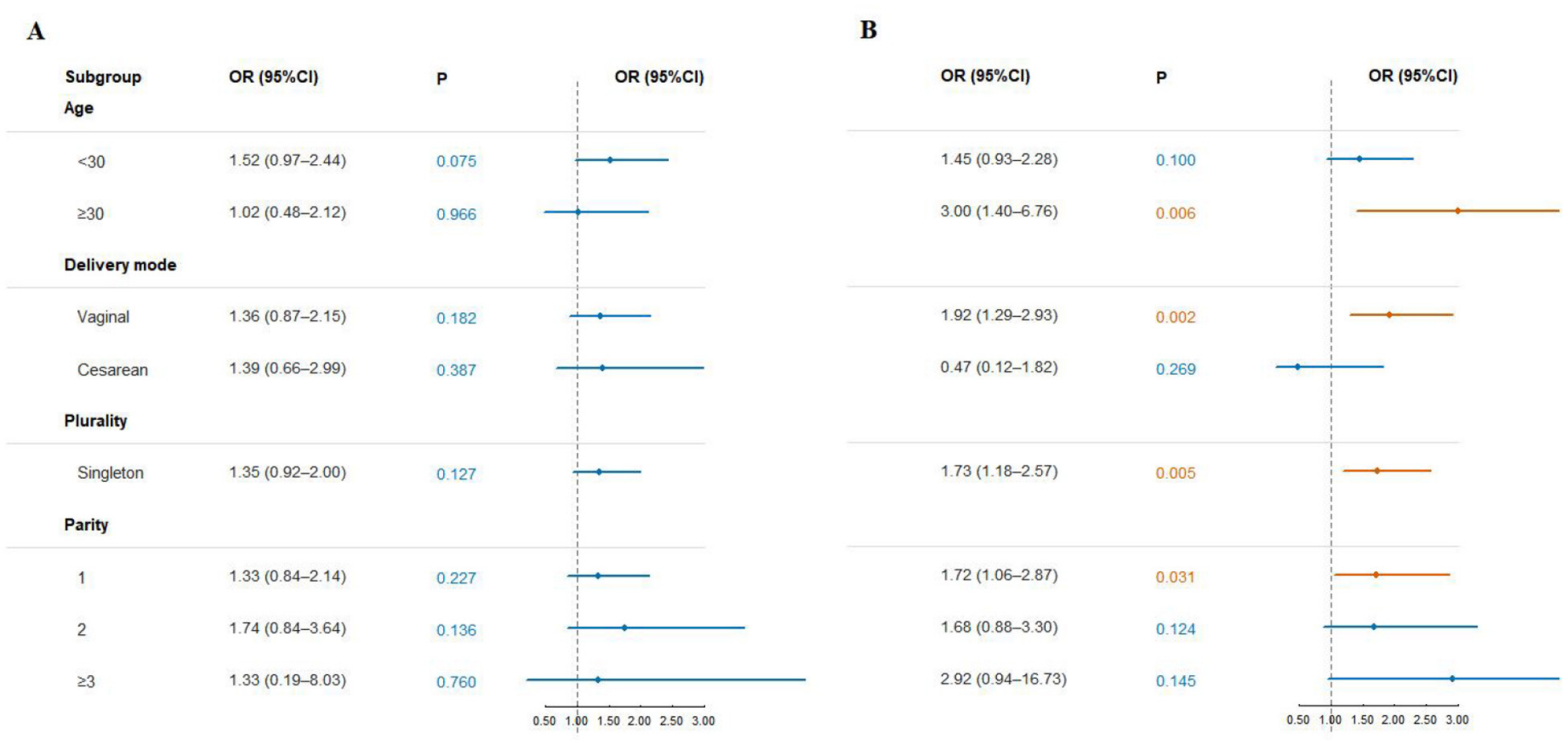
Forest plots illustrating subgroup analysis of BW and moderate-to-severe anterior/posterior vaginal wall prolapse. **(A)** Anterior vaginal wall. **(B)** Posterior vaginal wall.

For the anterior vaginal wall, the effects varied across subgroups, with most not reaching statistical significance; only women aged < 30 years showed a borderline association (OR = 1.52, 95% CI: 0.97–2.44, *P* = 0.075). In contrast, the associations for the posterior vaginal wall were more consistent. They remained significant in several subgroups, including women aged ≥ 30 years (OR = 3.00, 1.40–6.76, *P* = 0.006), those with vaginal delivery (OR = 1.92, 1.29–2.93, *P* = 0.002), singleton pregnancies (OR = 1.73, 1.18–2.57, *P* = 0.005), and women with parity = 1 (OR = 1.72, 1.06–2.87, *P* = 0.031). No significant associations were observed among women aged < 30 years, those with cesarean delivery, or those with parity ≥ 2 (Figure 3).

Overall, these findings showed that the relationship between higher BW and the risk of moderate-to-severe posterior vaginal wall prolapse was relatively consistent across different populations and clinical contexts. In contrast, the association with anterior wall prolapse was comparatively weaker.

### 3.5 Sensitivity analysis

To evaluate the robustness of the main findings, sensitivity analyses were conducted by restricting the study population to women with singleton pregnancies. The overall associations between BW and vaginal wall prolapse remained consistent with the primary analyses.

In the fully adjusted model, women in the highest BW quartile (≥3.415 kg) had a significantly increased risk of posterior vaginal wall prolapse compared with those in the lowest quartile (OR = 2.43, 95% CI: 1.51–3.96; *P* for trend < 0.001). For anterior vaginal wall prolapse, a similar direction of association was observed, with the highest BW quartile showing a significantly elevated risk (OR = 1.67, 95% CI: 1.06–2.66; *P* for trend = 0.0133) (Supplementary Table S5).

## 4 Discussion

### 4.1 Main findings

This study was a retrospective analysis based on medical records of postpartum women and employed a cross-sectional design to evaluate the association between neonatal BW and maternal anterior and posterior vaginal wall prolapse (POP-Q stage ≥ II). The results demonstrated that higher BW significantly increased the maternal risk of posterior vaginal wall prolapse, showing a precise dose–response relationship. To further assess the robustness of the primary findings with respect to outcome definition, POP-Q stage ≥ I was additionally examined as the outcome in sensitivity analyses (Supplementary Table S4). The overall direction of the associations was broadly consistent with the main analyses; however, because the prevalence of POP-Q stage ≥ I in our sample was close to universal, the ability to discriminate risk across BW groups was limited. Therefore, results for the POP-Q stage ≥ I are presented only as sensitivity analyses.

After full adjustment for age, delivery mode, plurality, and parity, each 1 kg increase in BW was associated with a 71% higher risk of posterior wall prolapse (OR = 1.71, 95% CI: 1.18–2.53, *P* = 0.006). Quartile analysis further confirmed that, compared with the lowest BW quartile, women in the highest BW group (≥3.415 kg) had a 2.26-fold higher risk (*P* for trend < 0.001). The RCS model revealed a non-linear pattern, with an inflection point around 3.7 kg. In contrast, the association between BW and anterior vaginal wall prolapse was relatively weaker. In the unadjusted and partially adjusted models, higher BW was significantly associated with increased anterior wall prolapse risk (*P* for trend < 0.05). After full adjustment, the overall trend attenuated but remained suggestive, with elevated risks observed in the moderate (3.15–3.415 kg) and higher (≥3.415 kg) BW groups (OR = 1.62, 95% CI: 1.02–2.61; OR = 1.53, 95% CI: 0.95–2.46). In addition, delivery mode showed a clear effect-modifying role, indicating that women who delivered vaginally were more likely to experience posterior wall support impairment when exposed to higher BW. Overall, this study identified high BW as an independent risk factor for maternal posterior vaginal wall prolapse after childbirth, while its association with anterior wall prolapse appeared comparatively limited.

### 4.2 Association between BW and anterior/posterior vaginal wall prolapse

Multivariate analysis in this study indicated that BW was significantly associated with the risk of posterior vaginal wall prolapse, while the association with anterior wall prolapse was relatively weaker. In the unadjusted model, anterior and posterior wall prolapse risks increased by approximately 51% and 76%, respectively. The upward trend in posterior wall risk remained stable even after progressively adjusting for potential confounders, including age, parity, delivery mode, and plurality. This finding suggests that the impact of fetal weight on pelvic floor support structures may be relatively independent, rather than solely mediated by the mode or duration of delivery. The underlying mechanism may be that greater BW exerts more substantial mechanical traction on pelvic floor supportive structures during vaginal delivery, potentially exceeding the physiological tolerance limit of pelvic ligaments (such as the cardinal and uterosacral ligaments) and fascial collagen fibers. This excessive stretch may lead to increased collagen degradation and loosening of connective tissues, accompanied by overdistension and microtrauma of the levator ani and other pelvic floor muscles. Such injuries are often irreversible, ultimately compromising the integrity of the pelvic support framework and markedly weakening its overall load-bearing capacity (14). Compared with the anterior wall, the posterior vaginal wall—located anterior to the rectum—endures dual traction forces from fetal head rotation and posterior compartment pressure, making it more susceptible to prolapse. In contrast, the anterior compartment is partly protected by the pubic symphysis and bladder base, which help disperse mechanical stress. This anatomical difference may explain why the association with anterior wall prolapse did not reach statistical significance in this study (15–17).

Age and parity are well-established core risk factors for POP. A systematic review and meta-analysis reported that the risk of primary POP increases by approximately 34% for every 10-year increment in age. At the same time, multiparity (≥2 vaginal deliveries) has been identified as a “confirmed risk factor” significantly elevating prolapse incidence (18). Regarding delivery mode, vaginal birth has been widely recognized as the most direct and dominant initiating factor for pelvic floor structural damage and dysfunction (19). MRI-based evidence has shown that 20–36% of women experience levator ani muscle avulsion or morphologic abnormalities after their first vaginal delivery, with substantially higher risks compared to cesarean delivery (20). Furthermore, an extensive prospective cross-sectional study of more than 1,500 women found that the risk of developing POP within 5–10 years postpartum was two to three times higher after vaginal delivery than after cesarean section (21). With regard to plurality, twin pregnancy has been proposed to increase prolapse risk through sustained elevation of intra-abdominal pressure and uterine distension, leading to prolonged stress exposure of the pelvic floor (22). Although twin pregnancies may impose a greater mechanical load on the pelvic floor, our sensitivity analyses restricted to singleton pregnancies yielded similar results, suggesting that the inclusion of twin births did not drive the main associations.

### 4.3 Threshold analysis between BW and anterior/posterior vaginal wall prolapse

RCS analysis further demonstrated a non-linear relationship between BW and the maternal risk of anterior and posterior vaginal wall prolapse (POP-Q ≥ II). For the anterior vaginal wall, the inflection point was approximately 3.2 kg; when BW was below this threshold, the risk increased significantly with higher BW (OR = 1.82, 95% CI: 1.02–3.22, *P* = 0.041), whereas above 3.2 kg, the association plateaued (*P* = 0.748). These results suggest a non-linear relationship between the risk of anterior vaginal wall prolapse and BW, with a significant increase in risk at approximately 3.2 kg (Q3 range), indicating that a specific threshold of BW may act as a risk inflection point. This threshold effect was not observed in other weight ranges. However, these findings should be interpreted with caution, as they may reflect limited data at extreme birthweights, and clinical decision patterns (e.g., cesarean for suspected macrosomia) might also influence the observed patterns. Although the overall continuous association was not significant, this finding highlights the importance of considering BW thresholds when assessing the risk of anterior vaginal wall prolapse. This threshold effect is crucial for understanding the complex relationship between BW and PFDs. For the posterior wall, the inflection point was approximately 3.7 kg; below this value, the risk rose steeply (OR = 2.20, 95% CI: 1.38–3.51, *P* < 0.001), but above 3.7 kg, the curve flattened (*P* = 0.586), indicating an apparent threshold effect. This non-linear pattern was similar to the findings of Du et al., who investigated the association between BW and postpartum UI among Chinese primiparous women. They also identified an inflection point around 3.9 kg, beyond which additional increases in BW no longer significantly elevated the risk of incontinence (95% CI: 0.76–1.21, *P* = 0.775) (23). This observation differs from previous studies (24, 25) that assumed a linear positive relationship between increasing BW and pelvic floor structural injury. The discrepancy may be related to a physiological stress-saturation effect of pelvic tissues: once the supportive structures (e.g., levator ani, fascial arcus tendineus) reach their maximal tensile threshold, the distribution of mechanical stress stabilizes, and further increases in fetal weight may impose load but exert diminished incremental damage (26). Moreover, some macrosomic fetuses may have undergone early obstetric intervention, such as elective cesarean section or assisted delivery (6), which could have mitigated the direct tractional damage to the pelvic floor. This may help explain why the upward risk slope attenuated in the higher BW range. This pattern suggests that the risk of pelvic floor injury is most sensitive within a moderate BW range and gradually plateaus once the corresponding thresholds are exceeded, consistent with the mechanical load-bearing properties and strain behavior of pelvic floor tissues during childbirth. This plateauing pattern differs markedly from the traditional assumption of a linear dose–response relationship, indicating that once fetal weight exceeds these thresholds, pelvic support structures may approach their stress-bearing capacity, such that further increases in fetal weight result only in diminishing incremental damage, reflecting a stress-saturation effect.

### 4.4 Clinical implications and future perspectives

This study reveals an independent, non-linear association between BW and maternal anterior and posterior vaginal wall prolapse, offering preliminary new insights into prenatal risk identification and perinatal management. BW is not only an important indicator of fetal development but also potentially reflects the mechanical load placed on the maternal pelvic floor during delivery. The results suggest a dose–response pattern between increasing fetal weight and postpartum prolapse risk, with the greatest risk sensitivity observed in the moderate-to-high BW range (approximately 3.2–3.7 kg). For the anterior vaginal wall, although the overall continuous association was not statistically significant, this finding emphasizes the importance of considering BW threshold effects when assessing prolapse risk. This threshold effect is crucial for understanding the complex relationship between BW and PFDs, and future research should further explore this to help develop more personalized clinical management strategies. For pregnant women with weak pelvic floor support, a history of vaginal delivery, or other high-risk factors, dynamic monitoring of fetal weight and individualized delivery strategies may have potential preventive value. By appropriately controlling weight gain during pregnancy and developing individualized delivery management strategies based on maternal and fetal risk assessment, it may help reduce the risk of early postpartum pelvic floor overload and anatomical descent. In addition, women who deliver larger babies should be included in postpartum follow-up and rehabilitation programs. Early screening for pelvic floor function and pelvic floor muscle training may help improve pelvic floor support during the recovery period and provide a foundation for future monitoring and intervention.

Furthermore, early postpartum POP-Q measurements need to be interpreted with caution. At this stage, the pelvic floor muscles, fascia, and connective tissues are still undergoing recovery (27). Therefore, the prolapse prevalence reported in this study should be understood as reflecting early postpartum pelvic support status rather than long-term stable POP outcomes. The POP-Q results at this stage may partly reflect transient physiological laxity rather than established prolapse, leading to a slight overestimation of early prolapse prevalence. Accordingly, the prolapse prevalence reported in this study should be interpreted as reflecting early postpartum pelvic support rather than the long-term outcomes of POP.

Finally, previous prospective studies have shown that moderate or more advanced anatomical prolapse (POP-Q ≥ II) assessed at 6 weeks postpartum may partially resolve over time. However, its occurrence is closely associated with pre-existing pelvic floor structural characteristics during pregnancy, suggesting that early POP-Q ≥ II may reflect an underlying structural predisposition rather than merely transient postpartum physiological changes (28). Therefore, it is recommended that women who deliver larger babies, particularly those with weak pelvic floor support, be included in postpartum follow-up and rehabilitation programs. Further multicenter prospective studies combining imaging and biomechanical parameters can quantitatively assess the impact of different BW thresholds on pelvic floor structural stress and explore the complex interactions between neonatal BW, delivery mode, and pelvic floor injury. This will help improve obstetric risk stratification models and provide evidence for establishing an individualized perinatal management system focused on maternal pelvic floor health.

## 5 Strengths and limitations

This study offers multiple innovations from both a research perspective and clinical significance. First, approaching the issue from the perspective of maternal–fetal interaction systematically revealed the independent effect of BW on pelvic floor supportive structure injury, emphasizing that BW is not only an indicator of neonatal outcome but also a key variable reflecting maternal delivery-related mechanical stress. Second, by analyzing anterior and posterior vaginal wall prolapse separately as outcome variables, the study is the first to propose that these compartments may have distinct biomechanical thresholds under fetal weight loading, suggesting differential tolerance to tensile stress across anatomical regions. Finally, by exploring the relationship among fetal weight, delivery mode, and pelvic floor injury, the study introduces the concept that the moderate-to-high BW range represents a critical window of pelvic floor vulnerability, providing evidence to guide individualized weight management and early preventive interventions in clinical practice.

The study also has several limitations. First, this study is a single-center retrospective study with a relatively small sample size, which may reduce the statistical power for detecting weaker associations, such as anterior vaginal wall prolapse, and limit the depth of subgroup analyses. Additionally, the findings may not be easily generalized to a broader Chinese population or other clinical settings. While most of the study participants were drawn from the routine postpartum pelvic floor checkup population at 42 days, the entire sample was sourced from the Pelvic Floor Rehabilitation Center at a tertiary maternal and child health hospital and includes a small number of patients who visited outside the standard postpartum window. This may introduce selection bias and limit the generalizability of the results to the general postpartum population at the community level. Secondly, although we adjusted for multiple important covariates, we could not completely exclude the influence of unmeasured confounding factors. The medical record system lacked several key obstetric and perinatal variables, such as pre-pregnancy BMI, gestational weight gain, duration of the second stage of labor, genetic predisposition affecting connective tissue mechanics, physical activity level, and further subdivision of instrumental deliveries (e.g., forceps delivery, which was the only type recorded in this study). These factors could potentially affect the relationship between birth weight and prolapse risk, and their exclusion may limit the generalizability of the study findings. Therefore, future research should consider including these variables in the analysis to better understand their impact on the observed relationships. Furthermore, although the medical records included information on perineal episiotomy, perineal laceration, and instrumental deliveries, and descriptive statistics were performed for these variables in the baseline characteristics, these variables are more likely to lie in the causal pathway between birth weight and pelvic floor damage. Including them in the multivariate model could lead to over-adjustment, so they were not included in the main regression analysis. Also, the incidence of instrumental deliveries in this study population was low, and the related exposure distribution was relatively concentrated. As a result, the findings mainly reflect characteristics of the population with a low proportion of instrumental deliveries and may not be directly extrapolated to populations with a higher rate of instrumental deliveries. Finally, the POP-Q assessment in this study was conducted in the early postpartum period, when the pelvic floor tissues are still in the physiological recovery process. Therefore, the observed prolapse symptoms may reflect transient changes rather than long-term, stable pelvic organ prolapse.

## 6 Conclusion

In conclusion, among Chinese postpartum women, higher BW was identified as an independent risk factor for moderate-to-severe posterior vaginal wall prolapse, exhibiting a precise dose–response relationship. The risk of prolapse significantly increases around a BW of 3.7 kg (posterior vaginal wall) and 3.2 kg (anterior vaginal wall), suggesting the presence of a threshold effect. Beyond these points, the impact of BW on prolapse risk stabilizes or slightly decreases, emphasizing the importance of considering BW thresholds when assessing prolapse risk. Although the overall continuous association did not reach statistical significance, this threshold effect is crucial for understanding the complex relationship between BW and PFDs.

These findings underscore the importance of prenatal nutritional counseling and weight management to optimize fetal size—not only as a means of improving perinatal outcomes but also as a potential strategy for minimizing risks to maternal pelvic floor health. Furthermore, targeted postpartum pelvic floor screening and intervention are recommended for women who deliver larger infants. Future studies should aim to expand sample size, incorporate biomechanical and genetic biomarkers, and conduct long-term follow-up to elucidate underlying mechanisms and validate the effectiveness of preventive measures.

## Data Availability

The original contributions presented in the study are included in the article/Supplementary material, further inquiries can be directed to the corresponding author.
