# Supplementary material for: Association between neonatal birthweight and maternal vaginal wall prolapse: a retrospective analysis of postpartum women
Source: Front Med (Lausanne). 2026 Mar 12;13:1792690. doi: 10.3389/fmed.2026.1792690 (PMC13017813; doi:10.3389/fmed.2026.1792690)
Supplement: Supplementary file 1 [file Supplementary_file_1.docx]

**Supplementary Figure S1. Schematic Diagram of Anatomical Points in the POP-Q Assessment System**


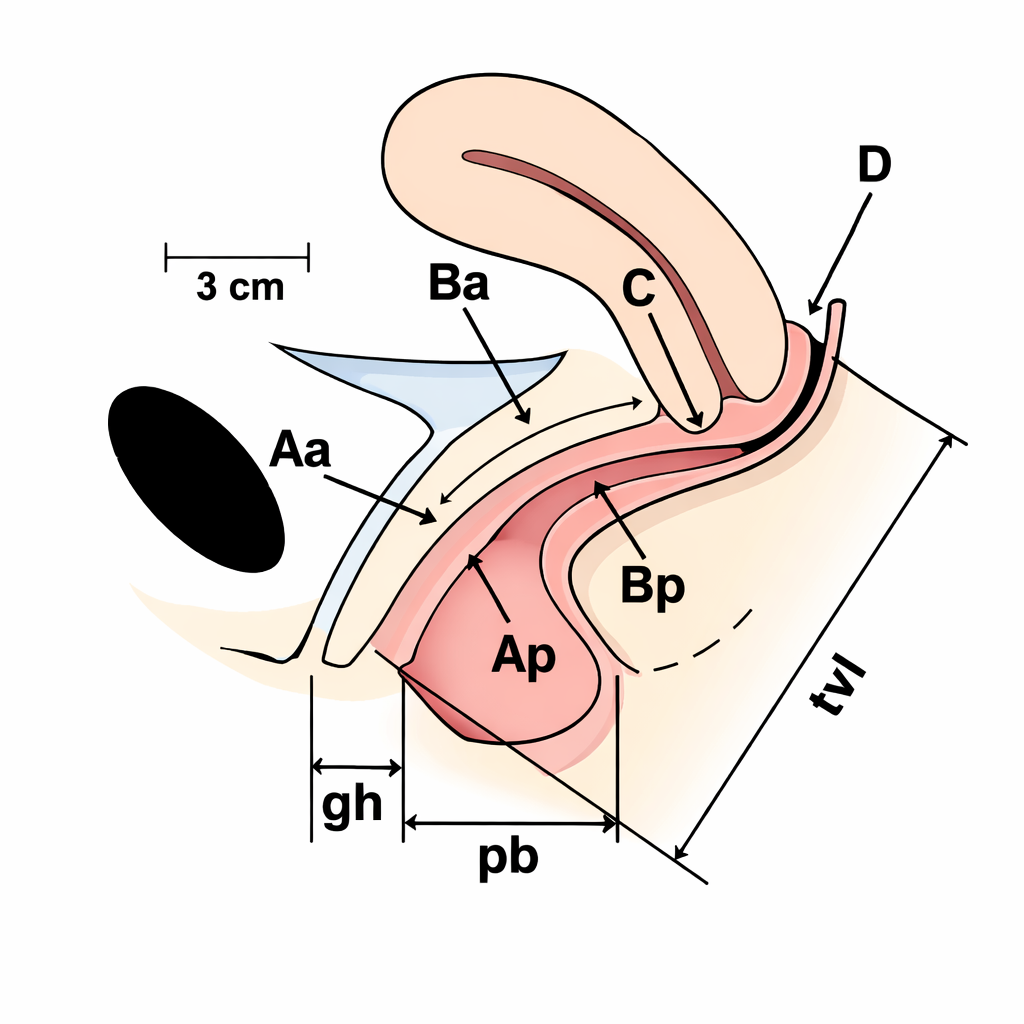


Note: The POP-Q system evaluates POP using nine standardized landmarks (Aa, Ba, C, D, Ap, Bp, gh, pb, tvl). Points Aa, Ba, and C represent the anterior vaginal wall; Ap, Bp, and D represent the posterior wall or cervix; gh denotes the genital hiatus, pb the perineal body length, and tvl the total vaginal length. The relative position of each point to the hymenal plane (in centimeters) determines the prolapse stage. Modified from Bump et al. (1996) **(1).**

**Supplementary Table S1. Core Measurement Indicators of the POP-Q System**

| Category | Anatomical Point / Parameter | Definition & Measurement Method | Normal Range / Significance |
| --- | --- | --- | --- |
| Anterior Prolapse | Point Aa | Located 3 cm proximal to the external urethral meatus in the midline of the anterior vaginal wall (corresponding to the "urethrovesical crease"); measured as the distance from the hymenal plane. | Normal range: -3 to +3 cm (< -3 cm indicates normal position; > +3 cm suggests inferior anterior vaginal wall prolapse) |
|  | Point Ba | The most distal (most dependent) point on the upper anterior vaginal wall, ranging from the vaginal cuff or anterior vaginal fornix to Point Aa. | Normal range: -3 cm (in the absence of prolapse); aligns with the position of the vaginal cuff in cases of total posthysterectomy vaginal eversion |
| Apical Prolapse | Point C | The most distal edge of the cervix (in women with an intact uterus) or the leading edge of the vaginal cuff scar (after total hysterectomy). | Normal position: -TVL to -(TVL - 2) cm (TVL = total vaginal length; e.g., if TVL = 10 cm, Point C should be between -10 cm and -8 cm) |
|  | Point D | Location of the posterior fornix (pouch of Douglas) in women with an intact cervix, reflecting the supportive function of the uterosacral ligaments. | Normal position: More proximal than Point C (e.g., if Point C = -8 cm, Point D = -10 cm); used to differentiate "cervical elongation" (Point C is more distal than Point D); omitted in women without a cervix |
| Posterior Prolapse | Point Ap | Located 3 cm proximal to the hymen in the midline of the posterior vaginal wall, symmetric to Point Aa on the anterior wall. | Normal range: -3 to +3 cm (> +3 cm suggests inferior posterior vaginal wall prolapse) |
|  | Point Bp | The most distal (most dependent) point on the upper posterior vaginal wall, ranging from the vaginal cuff or posterior vaginal fornix to Point Ap. | Normal range: -3 cm (in the absence of prolapse); aligns with the position of the vaginal cuff in cases of total posthysterectomy vaginal eversion |
| Length Parameters | Total Vaginal Length (TVL) | Maximum depth of the vagina after complete reduction of prolapse (excluding eccentrically elongated vaginal walls). | Normal range in adult women: ~8–12 cm; used to determine prolapse severity (e.g., Stage IV prolapse requires reference to TVL) |
|  | Genital Hiatus (gh) | Distance from the midpoint of the external urethral meatus to the posterior margin of the hymen; if the hymen is lax, the firm palpable tissue of the perineal body is used as the posterior margin. | Normal range: ~2–4 cm; hiatus enlargement (> 4 cm) indicates pelvic floor muscle support weakness |
|  | Perineal Body (pb) | Distance from the posterior margin of the genital hiatus to the midpoint of the anus. | Normal range: ~2–3 cm; shortening suggests perineal body injury (e.g., obstetric laceration) |

**Note: Adapted from Bump et al. (1996) (1).**

**Supplementary Table S2. POP Staging System Based on the POP-Q System**

| Stage | Core Definition (Based on the Most Distal Prolapsed Point) | Clinical Example |
| --- | --- | --- |
| 0 | No prolapse: Points Aa, Ap, Ba, and Bp are all -3 cm; Points C/D are between -TVL cm and -(TVL - 2) cm (e.g., if TVL = 10 cm, Point C ≤ -8 cm). | Normal pelvic floor status in healthy nulliparous women |
| I | Fails to meet Stage 0 criteria, but the most distal prolapsed point is > 1 cm above the hymenal plane (i.e., position < -1 cm). | Anterior wall Point Ba = -2 cm (2 cm above the hymen); no obvious clinical symptoms |
| II | The most distal prolapsed point is within ±1 cm of the hymenal plane (i.e., position ≥ -1 cm and ≤ +1 cm). | Cervical Point C = +0.5 cm (0.5 cm below the hymen); the patient may occasionally experience vaginal heaviness |
| III | The most distal prolapsed point is > 1 cm below the hymenal plane but does not extend beyond (TVL - 2) cm (i.e., position > +1 cm and < +(TVL - 2) cm). | If TVL = 10 cm, vaginal cuff Point C = +7 cm (does not reach 8 cm); prolapsed tissue protrudes noticeably beyond the vaginal introitus |
| IV | Complete eversion: The most distal prolapsed point is ≥ +(TVL - 2) cm (i.e., prolapse length is nearly equal to the total vaginal length). | Total posthysterectomy vaginal eversion, with Points Ba, Bp, and C all = +8 cm (TVL = 10 cm) |

**Note: Adapted from Bump et al. (1996) (1).**

**Supplementary Table S3. Evidence supporting inclusion of covariates in the multivariable models**

| Variable | Rationale for inclusion | Evidence type | References |
| --- | --- | --- | --- |
| Age | Advancing age is associated with progressive degeneration of pelvic support structures and increased POP prevalence, especially after age 50. | Global epidemiological analysis (GBD-based observational study) | (2) |
|  | POP prevalence increases markedly with age; clinical data show peak incidence between 60–69 years, highlighting aging as a major risk factor. | Retrospective clinical observational study | (3) |
| Parity | Each additional childbirth was associated with a progressively increased risk of POP; compared to one childbirth, having two, three, or ≥4 births increased the risk by 34%, 57%, and 104%, respectively. | Prospective cohort study | (4) |
|  | Higher parity (≥5 births) increased POP risk fivefold (AOR = 5.2, 95% CI: 2.2–12.6), confirming cumulative pelvic floor damage from repeated childbirth. | Case–control study | (5) |
| Mode of delivery | Vaginal childbirth was strongly associated with symptoms and signs of POP. Women with ≥1 vaginal delivery had over twice the prevalence of prolapse symptoms compared to nulliparas, confirming vaginal delivery as a major etiological factor. | Retrospective clinical observational study | (6) |
|  | Vaginal delivery markedly increased POP risk compared with cesarean section. | Systematic review and meta-analysis | (7) |
| Plurality | Twin pregnancy was identified as an independent risk factor for PFDs. Women with twin pregnancies had significantly higher rates of prolapse symptoms and ultrasound evidence of pelvic floor descent compared with singleton pregnancies. | Prospective cohort study | (8) |
| BW | Higher infant BW significantly increased the risk of POP, likely due to greater mechanical strain on the pelvic floor during vaginal delivery. | Prospective cohort study | (9) |

**Supplementary Table S4. Sensitivity analysis using POP-Q stage ≥ I as the outcome**

| Outcome | Model | OR (95% CI) | *P* | Q1  (<2.9 kg) | Q2  (2.9–3.15 kg) | Q3  (3.15–3.415 kg) | Q4  (≥3.415 kg) | *P* for trend |
| --- | --- | --- | --- | --- | --- | --- | --- | --- |
| **Anterior** | POP-Q ≥ I cases, n |  |  | 189 | 198 | 209 | 199 |  |
|  | Unadjusted | 5.27  (0.77–27.93) | 0.060 | Reference | — | — | — | 0.328 |
|  | Partially adjusted | 5.19  (0.76–27.77) | 0.064 | Reference | — | — | — | 0.332 |
|  | Fully adjusted | 6.41  (0.78–39.58) | 0.055 | Reference | — | — | — | 0.208 |
| **Posterior** | POP-Q ≥ I cases, n |  |  | 117 | 147 | 157 | 158 |  |
|  | Unadjusted | 0.93  (0.55–1.72) | 0.800 | Reference | — | — | — | 0.024* |
|  | Partially adjusted | 0.90  (0.53–1.68) | 0.717 | Reference | — | — | — | 0.025* |
|  | Fully adjusted | 0.80  (0.41–1.64) | 0.531 | Reference | — | — | — | 0.034* |
| The outcome was defined as POP-Q stage ≥ I for anterior or posterior vaginal POP. | | | | | | | | |
| ORs and 95% CIs were calculated from binary logistic regression models (Firth correction used when separation was detected, if available). | | | | | | | | |
| *P* for trend were estimated using the BW quartile as an ordinal variable. | | | | | | | | |
| Partially adjusted includes age and parity. | | | | | | | | |
| Fully adjusted additionally includes the delivery mode and plurality. | | | | | | | | |
| Due to the extremely small number of non-case observations within several BW quartiles, quartile-specific odds ratios were unstable and are therefore not presented. | | | | | | | | |
| **P* < 0.05. | | | | | | | | |

**Supplementary Table S5. Sensitivity analysis of BW quartiles in singleton pregnancies**

|  |  | BW (kg) | | BW quartiles OR (95% CI) | | | | |
| --- | --- | --- | --- | --- | --- | --- | --- | --- |
| Outcome | Model | OR  (95% CI) | *P* | Q1  (<2.9 kg) | Q2  (2.9–3.15 kg) | Q3  (3.15–3.415 kg) | Q4  (≥3.415 kg) | *P* for trend |
| **Anterior** | POP-Q ≥ II cases, n |  |  | 142 | 153 | 164 | 160 |  |
|  | Unadjusted | 1.59 (1.12–2.30) | 0.0113* | Reference | 1.51  (0.99–2.32) | 1.92  (1.29–2.85) | 1.75 (1.17–2.61) | 0.00474* |
|  | Partially adjusted | 1.58 (1.10–2.29) | 0.0142* | Reference | 1.49  (0.98–2.29) | 1.88  (1.26–2.80) | 1.80 (1.20–2.70) | 0.00394* |
|  | Fully adjusted | 1.48 (1.02–2.17) | 0.0401* | Reference | 1.37  (0.88–2.14) | 1.74  (1.11–2.76) | 1.67 (1.06–2.66) | 0.0133* |
| **Posterior** | POP-Q ≥ II cases, n |  |  | 35 | 49 | 56 | 68 |  |
|  | Unadjusted | 1.85 (1.29–2.70) | 0.00108* | Reference | 1.62  (1.00–2.64) | 1.89  (1.20–2.98) | 2.52 (1.64–3.88) | 0.00108* |
|  | Partially adjusted | 1.86 (1.28–2.73) | 0.00122* | Reference | 1.62  (0.99–2.66) | 1.75  (1.11–2.75) | 2.58 (1.67–3.99) | 0.000116* |
|  | Fully adjusted | 1.80 (1.23–2.68) | 0.00285* | Reference | 1.48  (0.90–2.46) | 1.61  (0.99–2.68) | 2.43 (1.51–3.96) | 0.00035* |
| The outcome was defined as POP-Q stage ≥ II for anterior or posterior vaginal POP. | | | | | | | | |
| ORs and 95% CIs were calculated from binary logistic regression models (Firth correction used when separation was detected, if available). | | | | | | | | |
| *P* for trend were estimated using the BW quartile as an ordinal variable. | | | | | | | | |
| Partially adjusted includes age and parity. | | | | | | | | |
| Fully adjusted additionally includes the delivery mode and plurality. | | | | | | | | |
| **P* < 0.05. | | | | | | | | |

**Supplementary Table S6. Missing Data Summary of Study Variables (N = 864)**

| **Variable** | **Valid (N)** | **Missing (N)** | **Missing (%)** |
| --- | --- | --- | --- |
| **Age** | 864 | 0 | 0.0% |
| **Parity** | 853 | 11 | 1.3% |
| Plurality | 858 | 6 | 0.7% |
| **Delivery Mode** | 846 | 18 | 2.1% |
| **Urinary Incontinence** | 862 | 2 | 0.2% |
| **Chronic Pelvic Pain** | 862 | 2 | 0.2% |
| **Episiotomy** | 863 | 1 | 0.1% |
| **Perineal Laceration** | 864 | 0 | 0.0% |
| **Instrumental Delivery** | 864 | 0 | 0.0% |
| BW | 864 | 0 | 0.0% |

**References**

1. Bump RC, Mattiasson A, Bø K, Brubaker LP, DeLancey JO, Klarskov P, et al. The Standardization of Terminology of Female Pelvic Organ Prolapse and Pelvic Floor Dysfunction. *American journal of obstetrics and gynecology*. (1996) 175(1):10-7. doi: [10.1016/S0002-9378(96)70243-0](https://doi.org/10.1016/S0002-9378(96)70243-0)

2. Wang B, Chen Y, Zhu X, Wang T, Li M, Huang Y, et al. Global Burden and Trends of Pelvic Organ Prolapse Associated with Aging Women: An Observational Trend Study from 1990 to 2019. *Frontiers in public health*. (2022) 10:975829. doi: 10.3389/fpubh.2022.975829

3. Śliwa J, Rosner-Tenerowicz A, Kryza-Ottou A, Ottou S, Wiatrowski A, Pomorski M, et al. Analysis of Prevalence of Selected Anamnestic Factors among Women with Pelvic Organ Prolapse. *Advances in Clinical and Experimental Medicine*. (2018) 27(2):179-84. doi: 10.17219/acem/68994.

4. Brülle A-L, Wu C, Rasch V, Simonsen MK, Schøyen IS, Dahl C, et al. How Do Reproductive History and Anthropometry in Midlife Relate to Later Risk of Pelvic Organ Prolapse? A Prospective Cohort Study. *International Urogynecology Journal*. (2022) 33(12):3373-80. doi: 10.1007/s00192-022-05122-8

5. Borsamo A, Oumer M, Worku A, Asmare Y. Associated Factors of Pelvic Organ Prolapse among Patients at Public Hospitals of Southern Ethiopia: A Case-Control Study Design. *PloS one*. (2023) 18(1):e0278461. doi: 10.1371/journal.pone.0278461

6. Dietz HP, Rozsa D, Subramaniam N, Friedman T. Does Vaginal Parity Alter the Association between Symptoms and Signs of Pelvic Organ Prolapse? *Journal of Ultrasound in Medicine*. (2021) 40(4):675-9. doi: 10.1002/jum.15437

7. Larsudd-Kåverud J, Gyhagen J, Åkervall S, Molin M, Milsom I, Wagg A, et al. The Influence of Pregnancy, Parity, and Mode of Delivery on Urinary Incontinence and Prolapse Surgery—a National Register Study. *American journal of obstetrics and gynecology*. (2023) 228(1):61. e1-. e13. doi: 10.1016/j.ajog.2022.07.035

8. Zhou Y, Luo Y, Zhou Q, Xu J, Tian S, Liao B. Effect of Gestational Weight Gain on Postpartum Pelvic Floor Function in Twin Primiparas: A Single-Center Retrospective Study in China. *BMC Pregnancy and Childbirth*. (2023) 23(1):273. doi:  [10.1186/s12884-023-05602-9](https://doi.org/10.1186/s12884-023-05602-9)

9. Hendrix SL, Clark A, Nygaard I, Aragaki A, Barnabei V, McTiernan A. Pelvic Organ Prolapse in the Women's Health Initiative: Gravity and Gravidity. *American journal of obstetrics and gynecology*. (2002) 186(6):1160-6. doi: [10.1067/mob.2002.123819](https://doi.org/10.1067/mob.2002.123819)
